# Evaluating dry vs. wet disinfection in boot baths on detection of porcine epidemic diarrhea virus and porcine reproductive and respiratory virus RNA

**DOI:** 10.1093/tas/txac150

**Published:** 2022-11-12

**Authors:** Olivia L Harrison, Grace E Houston, Allison K Blomme, Haley K Otott, Jianfa Bai, Elizabeth G Poulsen Porter, Jason C Woodworth, Chad B Paulk, Jordan T Gebhardt, Cassandra K Jones

**Affiliations:** Department of Animal Sciences and Industry, Kansas State University, Manhattan, KS, USA; Department of Diagnostic Medicine/Pathobiology, Kansas State University, Manhattan, KS, USA; Department of Grain Science and Industry, Kansas State University, Manhattan, KS, USA; Department of Grain Science and Industry, Kansas State University, Manhattan, KS, USA; Department of Diagnostic Medicine/Pathobiology, Kansas State University, Manhattan, KS, USA; Department of Diagnostic Medicine/Pathobiology, Kansas State University, Manhattan, KS, USA; Department of Animal Sciences and Industry, Kansas State University, Manhattan, KS, USA; Department of Grain Science and Industry, Kansas State University, Manhattan, KS, USA; Department of Diagnostic Medicine/Pathobiology, Kansas State University, Manhattan, KS, USA; Department of Animal Sciences and Industry, Kansas State University, Manhattan, KS, USA

**Keywords:** boot bath, PEDV, PRRSV, swine

## Abstract

Maintaining biosecurity between swine barns is challenging, and boot baths are an easily implementable option some utilize to limit pathogen spread. However, there are concerns regarding their efficacy, especially when comparing wet or dry disinfectants. The objective of this study was to evaluate the efficacy of boot baths in reducing the quantity of detectable porcine epidemic diarrhea virus (**PEDV**) and porcine reproductive and respiratory syndrome virus (**PRRSV**) genetic material using wet or dry disinfectants. Treatments included 1) control, 2) dry chlorine powder (Traffic C.O.P., PSP, LLC, Rainsville, AL), and 3) wet quaternary ammonium/glutaraldehyde liquid (1:256 Synergize, Neogen, Lexington, KY). Prior to disinfection, rubber boots were inoculated with 1 mL of a co-inoculants of PRRSV (1 × 10^5^ TCID_50_ per mL) and PEDV (1 × 10^5^ TCID_50_ per mL) and dried for 15 min. After the drying period, a researcher placed the boot on the right foot and stepped directly on a stainless steel coupon (control). Alternatively, the researcher stepped first into a boot bath containing either the wet or dry sanitizer, stood for 3 s, and then stepped onto a steel coupon. After one minute, an environmental swab was then collected and processed from each boot and steel coupon. The procedure was replicated 12 times per disinfectant treatment. Samples were analyzed using a duplex qPCR at the Kansas State Veterinary Diagnostic Laboratory. Cycle threshold values were analyzed using SAS GLIMMIX v 9.4 (SAS, Inc., Cary, NC). There was no evidence of a disinfectant × surface × virus interaction (*P* > 0.10). An interaction between disinfectant × surface impacted (*P* < 0.05) the quantity of detectable viral RNA. As expected, the quantity of the viruses on the coupon was greatest in the control, indicating that a contaminated boot has the ability to transfer viruses from a contaminated surface to a clean surface. Comparatively, the dry disinfectant treatment resulted in no detectable viral RNA on either the boot or subsequent coupon. The wet disinfectant treatment had statistically similar (*P* > 0.05) viral contamination to the control on the boot, but less viral contamination compared to the control on the metal coupon. In this experiment, a boot bath with dry powder was the most efficacious in reducing the detectable viral RNA on both boots and subsequent surfaces.

## INTRODUCTION

Disease spread between populations of animals is a major concern for many swine producers. Protocols like changing clothes and wearing plastic boot covers help reduce farm-to-farm disease spread between production sites (Otake et al., 2002; Dee et al., 2004). However, room-to-room disease spread within a single production site is typically limited due to challenges in infrastructure and practicality of implementation. One easily implemented option is to place a boot bath between rooms with the intent to sanitize the boot bottoms of personnel as they move from one room to another.

The efficacy of the boot bath in a production system is dependent on the disinfectant utilized, the pathogen of concern, and the maintenance of the system itself. Muckey et al. (2021) saw that surface type largely affected the ability of different disinfectants to reduce the presence of porcine epidemic diarrhea virus (**PEDV**). As expected, smooth surfaces are easier to clean than textured ones, such as rubber boots, because the texture requires additional steps to remove organic matter for effective sanitation (Amass et al., 2000; Huss et al., 2017).

Boot baths have been demonstrated to be effective at preventing fomite transmission of porcine reproductive and respiratory disease syndrome (**PRRSV**), highly pathogenic and low pathogenic avian influenza, and aerobic bacteria (Dee et al., 2004; Hauck et al., 2017; Nasr et al., 2018). However, their usefulness is often questioned due to the maintenance needed to maintain efficacy (Amass et al., 2000, 2001; Bashandy et al., 2017). Historically, most boot baths have contained wet sanitizer, which can pose a slip hazard and quickly accumulates organic matter, potentially reducing its efficacy over time. Alternate dry powder disinfectants have recently become available, but there is little data to compare the efficacy of the dry powder compared to the wet disinfectant. Therefore, the objective of this study was to evaluate the efficacy of boot baths, using either wet or dry disinfectants, on the detectability of PEDV and PRRSV genetic material.

## MATERIALS AND METHODS

### Experimental Design

All experimental procedures were approved by the Institutional Biosafety Committee at Kansas State University (IBC **#**1511) and were conducted in the Cargill Feed Safety Research Center (FSRC) at the Kansas State University O.H. Kruse Feed Technology Innovation Center in Manhattan, KS.

### Preparation of Inoculum

Prior to the experiment, 4 mL of 1.33 × 10^6^ TCID_50_ per mL PEDV (USA/Co/2013) and 4 mL of 1.33 × 10^6^ TCID_50_ per mL PRRSV (wild type 1-7-4) were individually diluted with 36 mL phosphate buffer solution (PBS) in separate 50 mL conical tubes for an approximate final concentration of 1 × 10^5^ TCID_50_ per mL. Viruses were further divided into 10-mL aliquots and stored at −80 °C until the start of the experiment. The inoculants were tested via PCR at the time of sample analysis to validate the presence of viral RNA prior to disinfectant exposure.

### Preparation of Surfaces

At the start of the experiment, aliquots of each virus were thawed at room temperature in a biosafety cabinet within the BSL-2 facility. Next, 9 mL of each virus was combined in a single container and gently agitated to create a single container with 18 mL of a PEDV/PRRSV co-inocula. From this container, 1-mL aliquots were drawn into individual 2-mL Monject syringes and stored in the biosafety cabinet for a maximum of 15 min.

Meanwhile, boots, boot baths, and surfaces were prepared within the BSL-2 facility but outside the biosafety cabinet. Thirty-six boots (size 12, right foot only; Tingley, Tingley Rubber Company, Piscataway, NJ) were placed upside down on a boot drying rack and dusted with approximately 2 g of autoclaved ground corn (600 microns) to disrupt the rubber surface tension of the boot prior to viral inoculation. Twenty-four plastic containers (35.5 cm × 25.4 cm × 10.2 cm; Van Ness Small Liter Pan, Van Ness Pets, Clifton, NJ) were filled with approximately 2.5 cm of either dry disinfectant (Traffic C.O.P., PSP, LLC, Rainsville, AL) or wet disinfectant (Synergize, Neogen, Lexington, KY). The dry disinfectant was a dry powder containing chlorine, silicates, and acid-impregnated zeolites used directly as received from the manufacturer. The wet disinfectant was a quaternary ammonium/glutaraldehyde liquid that required 1:256 dilution with water per the manufacturer’s directions. Thirty-six stainless steel coupons (10 cm × 10 cm; Built So-Well, LLC, Manhattan, KS) were autoclaved and placed at least 10 cm apart from one another.

### Surface Inoculation

One mL of the co-inoculant was distributed in the same location across the sole of each boot in two even lines. Boots were then allowed to air-dry for 15 min at room temperature (approx. 22 to 25 °C). After the drying period, a single designated researcher placed the boot on the right foot and stepped directly on a stainless-steel coupon (control). Alternatively, the researcher stepped first into a dry or wet boot bath and stood for 3 s before stepping onto the steel coupon. Boots were then placed back on the drying rack and surfaces were allowed to air-dry for 1 min at ambient temperature and humidity. Next, an environmental swab was collected and processed from each boot and steel coupon using procedures described by Elijah et al. (2021). Briefly, a 10 cm × 10 cm surgical cotton gauze swab was premoistened with PBS and stored in a 50-mL conical tube prior to the experiment. During sampling, the gauze was aseptically removed and swabbed across the sampling surface in an approximately 10 cm × 10 cm area before the swab was returned to the conical tube. Swabs were returned to the biosafety cabinet, where 20 mL of PBS was added to the conical tube. Conical tubes were then inverted for 10 to 15 s and incubated at room temperature for 1 h. After incubation, 1.75 mL of supernatant was aliquoted into cryovials. Upon exiting the facility, samples were placed in a −80 °C freezer until analysis. Other than temperature to slow viral degradation, no additional stop agents were used to end the disinfectant process. These procedures were repeated 12 times. Altogether, 72 environmental swabs were collected, representing 12 replicates of 3 boot bath treatments (control, dry disinfectant, or wet disinfectant) and two surfaces (rubber boot and stainless-steel coupon).

### Quantitative Viral Analysis

Environmental swabs were analyzed for quantitative real-time polymerase chain reaction (qRT-PCR) for PEDV and PRRSV at the Kansas State University Veterinary Diagnostic Laboratory using procedures similar to those described by Elijah et al. (2022). First, 50 µL of supernatant was placed in a deep well plate and RNA was extracted using a Kingfisher Flex magnetic particle processor (Fisher Scientific, Pittsburgh, PA) and a MagMAX-96 Viral Isolation Kit (Life Technologies, Grand Island, NY). The final elution volume was reduced to 60 µL, and extracted RNA was stored at −80 °C until analyzed for PEDV or PRRSV using a qRT-PCR duplex assay with a maximum cycle threshold of 45. Results were reported as the number of samples considered PCR positive and the cycle threshold (Ct) at which either PEDV or PRRSV RNA was detected.

### Statistical Analysis

Results were analyzed as a split plot design with boot bath pan as the main experimental unit and surface (either boot or coupon) as the sub-plot using the GLIMMIX procedure of SAS version 9.4 (SAS Institute Inc., Cary, NC). Fixed effects included disinfectant (control, dry, or wet), surface type (boot or steel), virus (PEDV or PRRSV), and their associated interactions. Random effect included boot bath pan. There were two response criteria considered, the proportion of PCR-positive samples and the quantity of detectable viral RNA. To estimate the proportion of PCR-positive samples, the number of samples with detectable PEDV or PRRSV RNA was placed in ratio to the number of total samples. Data were analyzed by fitting to a binary distribution, logit link, Laplace approximation, and ridge-stabilized Newton-Raphson algorithm. As a binary distribution model, data were fit by each individual interaction, starting with the disinfectant × surface type × virus interaction, and their subsequent main effects. To estimate the quantity of detectable viral RNA, the Ct of each sample was used. If no PEDV or PRRSV RNA was detected, samples were assigned a value of 45.0. A Kenward–Roger denominator degree of freedom adjustment was used, as well as a Tukey–Kramer multiple comparison adjustment. Results were considered significant at *P* ≤ 0.05.

## RESULTS AND DISCUSSION

There was no evidence of a disinfectant × surface × virus interaction (*P* > 0.05) for either the proportion of positive samples or their quantity of detectable viral RNA (Table 1). However, there was a disinfectant × surface interaction (*P* < 0.05) for both response criteria. There was no evidence (*P* > 0.05) that the proportion of PCR-positive samples differed between samples collected from boots or steel coupons for the control treatment or the boots for the wet disinfectant treatment. However, these all had a greater (*P* < 0.05) proportion of PCR-positive samples than the steel surface after the boot bath with wet disinfectant. There were no PCR-positive samples for either the boot or steel surface after the boot bath with dry disinfectant. The quantity of viral RNA was greater (*P* < 0.05) for the boots and steel coupons from the control treatment and the boots from the wet disinfectant treatment as compared to the steel coupons from the wet disinfectant treatment and either surface from the dry disinfectant treatment (Table 1).

**Table 1.** Detection of viral RNA on boots or subsequent steel surfaces after stepping in a boot bath containing a wet or dry disinfectant^*^

Item	Boot bath disinfectant type		
	Control	Dry	Wet
PCR positive^†^			
Boot	19/24^c^	0/24^a^	21/24^c^
Steel	22/24^c^	0/24^a^	9/24^b^
Ct^‡^			
Boot	37.0^c^	45.0^a^	38.1^c^
Steel	34.0^d^	45.0^a^	42.2^b^

^*^Boots were inoculated with 1 mL of a PEDV/PRRSV co-inoculant and were randomly subjected to one of three boot bath disinfectants. Boots were stepped onto a stainless-steel coupon (10 × 10 cm) after submersion in the boot bath. The dry disinfectant was a powder containing chlorine, silicates, and acid-impregnated zeolites (Traffic C.O.P., PSP LLC, Rainsville, AL). The wet disinfectant was liquid quaternary ammonia and glutaraldehyde blend (Synergize, Neogen, Lexington, KY). Samples with no detectable RNA were assigned a Ct value of 45.0. Disinfectant × surface × virus, *P* > 0.05.

^†^PCR positive: disinfectant × Surface, *P* = 0.0154.

^‡^Ct is the average cycle threshold value for both PEDV and PRRSV. Disinfectant × Surface, *P* = 0.0001; SEM = 0.60674.

^a,b,c,d^Means with differing superscripts differ significantly.

These results are similar to those reported by Hauck et al. (2017), which determined a dry chlorine-based disinfectant had greater efficacy at sanitizing high or low pathogenic avian influenza virus compared to either a wet quaternary ammonium/glutaraldehyde disinfectant or a control. Rabbe et al. (2012) also observed a reduced proportion of PRRSV-positive sample when dry disinfectant was used compared to wet disinfectant in the presence of fecal matter. However, in the absence of fecal matter, our results are contrary to Rabbe et al. (2012) as dry disinfectant had a greater proportion of PRRSV-positive samples as compared to wet disinfectant. One possible explanation for this discrepancy is that the active ingredients in the dry disinfectant used by Rabbe et al. (2012) were reported as phosphates, silicates, copper, and iron, whereas the current study utilized a product with sodium sulfate, sodium chloride, calcium hypochlorite, calcium chlorate, calcium chloride, calcium hydroxide, and calcium carbonate.

The observed differences across surface types are consistent with those previously reported (Huss et al., 2017; Muckey et al., 2021). It is important to reiterate that the rubber boot, but not the stainless-steel coupon, was directly inoculated with PEDV and PRRSV. Therefore, any viral RNA present on the steel surface was transferred from the boot. Dee et al. (2004) reported similar results, where surfaces that were affected by cross-contamination had less detectable viral RNA than those that were experimentally inoculated. Huss et al. (2017) identified smooth rubber as an ideal candidate for equipment that can easily be decontaminated, but the crevices of boots in this study may prevent fully effective disinfection. Muckey et al. (2021) also observed differences in detectable PEDV RNA between surface types when quaternary ammonium/glutaraldehyde was used as a disinfectant. In both Huss et al. (2017) and Muckey et al. (2021), however, the authors compared disinfectants on varying surface types that had been directly inoculated. Whereas this study only directly inoculated the rubber boot and relied on cross-contamination to spread the virus to the steel surface.

In addition to the disinfectant × surface interaction reported above, the quantity of detected viral RNA in this study was also affected by a disinfectant × virus interaction (*P* < 0.05; Table 2). Specifically, there were greater (*P* < 0.05) quantities of PEDV detected in the control samples than of PRRSV in the control or PEDV in samples from the boot bath with wet disinfectant. Again, no PEDV or PRSSV was detected in samples from the boot bath with dry disinfectant.

**Table 2.** Detection of PEDV and PRRSV RNA after stepping in a boot bath containing a wet or dry disinfectant^*^

Item	Boot bath disinfectant type		
	Control	Dry	Wet
PCR positive^†^			
PEDV	20/24	0/24	19/24
PRRSV	21/24	0/24	11/24
Ct^‡^			
PEDV	34.0^d^	45.0^a^	38.0^c^
PRRSV	37.0^c^	45.0^a^	42.3^b^

^*^Boots were inoculated with 1 mL of a PEDV/PRRSV co-inoculant and were randomly subjected to one of three boot bath disinfectants. Boots were stepped onto a stainless-steel coupon (10 × 10 cm) after submersion in the boot bath. The dry disinfectant was a powder containing chlorine, silicates, and acid-impregnated zeolites (Traffic C.O.P., PSP LLC, Rainsville, AL). The wet disinfectant was liquid quaternary ammonia and glutaraldehyde blend (Synergize, Neogen, Lexington, KY). Samples with no detectable RNA were assigned a Ct value of 45.0. Disinfectant × surface × virus, *P* > 0.05.

^†^PCR positive: disinfectant × virus, *P* > 0.05.

^‡^Ct is the average cycle threshold value for both boot and stainless-steel surfaces. Disinfectant × virus, *P* = 0.0019; SEM = 0.6574.

^a,b,c,d^Means with differing superscripts differ significantly.

Our results indicate using a wet disinfectant was able to reduce both PEDV and PRRSV viral RNA, but not to the extent observed with the dry disinfectant. Few studies have compared sanitizer efficacy in PEDV and PRRSV simultaneously. Elijah et al. (2022) evaluated sanitizer efficacy on vehicle interior surfaces and reported that surface type and sanitizer both impacted the proportion of positive PEDV- or PRRSV-positive samples. However, all the surfaces in the Elijah et al. (2022) experiment were inoculated directly, so it is probable that the initial Ct values of each virus is driving this interaction.

## LIMITATIONS

One of the major challenges of using boot baths is the buildup of organic matter during use, which may impact sanitizer efficacy. This project initially intended to include an organic matter (an equal combination of soil and fecal matter) component; however, the organic matter results had to be omitted because the particles in the organic matter interfered with the qRT-PCR assay. Furthermore, these results only report the quantity of detected viral RNA as determined by Ct, not the infectivity of these samples. It is hypothesized that chemical sanitizers are effective for PEDV or PRRSV because the sanitizer disrupts the viral envelope and prevents fomite-based transmission of infectious particles. This would likely result in one of two options: the fragments of viral RNA can be detected by qRT-PCR but are no longer capable of causing infection or the viruses can cause infection, but the viral particles present were below infectious dosages. Response criteria that evaluates infectivity, such as a bioassay or cell culture virus isolation assay, are necessary to draw full conclusions. However, others have reported that the reduction of viral RNA is indicative of infectivity, and so while these results are limited, they are nevertheless important in better understanding practical biosecurity measures. Continued research is necessary to improve both the practicality and efficacy of on-farm biosecurity measures, as well as the laboratory procedures used to test, apply, and interpret supported results.

## CONCLUSIONS

Boot baths are an easily-implemented biosecurity measure to reduce room-to-room viral transfer on swine farms and other facilities. A boot bath containing a dry chlorine powder in this experiment surpassed the performance of a boot bath containing a wet quaternary ammonium/glutaraldehyde liquid disinfectant. While the wet disinfectant reduced the quantity of viral RNA compared to the control, it did not reduce viral RNA of either virus beyond detectable limits. Future research should focus on the utilization of dry disinfectant in production settings and in the presence of organic matter, as well as to evaluate viral infectivity.

## References

[CIT0001] Amass, S. F., D. Ragland, and P. Spicer. 2001. Evaluation of the efficacy of a peroxygen compound, Virkon S, as a boot bath disinfectant. J. Swine Health Prod. 9:121–123.

[CIT0002] Amass, S. F., B. D. Vyverberg, D. Ragland, C. A. Dowell, C. D. Anderson, J. H. Stover, and D. J. Beaudry. 2000. Evaluating the efficacy of boot baths in biosecurity protocols. J. Swine Health Prod. 8:169–173.

[CIT0003] Bashandy, E. Y., S. A. Nasef, S. A. E. Nasr, M. F. Abdel-Aty, and O. M. K. Zahran. 2017. Efficacy of a novel foot pan in biosecurity protocols for control of salmonellae in poultry farms. J. Vet. Med. Res. 24:28–40. doi:10.21608/jvmr.2017.43260

[CIT0004] Dee, S., J. Deen, and C. Pijoan. 2004. Evaluation of 4 intervention strategies to prevent the mechanical transmission of porcine reproductive and respiratory syndrome virus. Can. J. Vet. Res. 68:19–26. 14979431 PMC1142125

[CIT0005] Elijah, C. G., C. K. Jones, C. Evans, H. K. Wecker, C. R. Stark, J. Bai, E. G. Poulsen-Porter, A. K. Blomme, J. C. Woodworth, C. B. Paulk, et al. 2022. Quantification of decontamination strategies for semi-truck cabs. In: Proceedings of the 53rd AASV Annual Meeting. p. 37–40. doi: 10.54846/am2022/s5-04

[CIT0006] Elijah, C. G., J. D. Trujillo, C. K. Jones, N. N. Gaudreault, C. R. Stark, K. R. Cool, C. B. Paulk, T. Kwon, J. C. Woodworth, I. Morozov, et al. 2021. Evaluating the distribution of African swine fever virus within a feed mill environment following manufacture of inoculated feed. PLoS One 16:e0256138. doi:10.1371/journal.pone.0256138 34383843 PMC8360541

[CIT0007] Hauck, R., B. Crossley, D. Rejmanek, H. Zhou, and R. A. Gallardo. 2017. Persistence of Highly Pathogenic and Low Pathogenic Avian Influenza Viruses in Footbaths and Poultry Manure. Avian Dis. 61:64–69. doi:10.1637/11495-091916-Reg 28301246

[CIT0008] Huss, A. R., L. L. Schumacher, R. A. Cochrane, E. Poulsen, J. Bai, J. C. Woodworth, S. S. Dritz, C. R. Stark, and C. K. Jones. 2017. Elimination of porcine epidemic diarrhea virus in an animal feed manufacturing facility. PLoS One 12:e0169612. doi:10.1371/journal.pone.0169612 28099453 PMC5242487

[CIT0009] Muckey, M. B., C. K. Jones, J. C. Woodworth, C. B. Paulk, S. S. Dritz, and J. T. Gebhardt. 2021. Using environmental sampling to evaluate the effectiveness of decontamination methods to reduce detection of porcine epidemic diarrhea virus RNA on feed manufacturing surfaces. Transl. Anim. Sci. 5:1–9. doi:10.1093/tas/txab121 PMC8346718 34377952

[CIT0010] Nasr, S. A. E., E. Ismael, S. E. Laban, E. M. Ismail, M. M. Hamoud, M. M. Zaki, and O. M. K. Zahran. 2018. Effectiveness of some disinfectants commonly used in footbaths inside poultry farms. ISOR J. Agri. Vet. Sci. 11:1–6. doi:10.9790/2380-1109020106.

[CIT0011] Otake, S., S. A. Dee, K. D. Rossow, J. Deen, H. S. Joo, T. W. Molitor, and C. Pijoan. 2002. Transmission of porcine reproductive and respiratory syndrome virus by fomites (boots and coveralls). J. Swine Health Prod. 10:59–65..

[CIT0012] Rabbe, C., D. Murray, and A. Sponheim. 2012. An evaluation of Stalosan F powder for deactivation of PRRSv. Proc. AASV. 2012:97–100.

